# Patient Preferences and Priorities for the Design of an Acute Kidney Injury Prevention Trial: Findings from a Consensus Workshop

**DOI:** 10.34067/KID.0000000000000554

**Published:** 2024-08-15

**Authors:** Meghan J. Elliott, Kirsten M. Fiest, Shannan Love, Dale Birdsell, Maureena Loth, Heather Dumka, Benny Rana, Nusrat Shommu, Eleanor Benterud, Sarah Gil, Dilaram Acharya, Tyrone G. Harrison, Neesh Pannu, Matthew T. James

**Affiliations:** 1 Department of Medicine, University of Calgary, Calgary, Alberta, Canada; 2 Department of Community Health Sciences, University of Calgary, Calgary, Alberta, Canada; 3 Department of Critical Care Medicine, University of Calgary, Calgary, Alberta, Canada; 4 Nephrology Research Group, University of Calgary, Calgary, Alberta, Canada; 5 Department of Medicine, University of Alberta, Edmonton, Alberta, Canada

**Keywords:** AKI, nephrology

## Abstract

**Key Points:**

- For AKI prevention trial recruitment, patients prioritized technology enabled prescreening and involvement of family members in the consent process.
- For trial intervention delivery, participants prioritized measures to facilitate ease of trial intervention administration and return visits.
- For AKI prevention trial outcomes, patient participants identified effects on kidney-related and other clinical outcomes as top priorities.

**Background:**

High-quality clinical trials are needed to establish the efficacy and safety of novel therapies for AKI prevention. In this consensus workshop, we identified patient and caregiver priorities for recruitment, intervention delivery, and outcomes of a clinical trial of cilastatin to prevent nephrotoxic AKI.

**Methods:**

We included adults with lived experience of AKI, CKD, or risk factors of AKI (*e.g*., critical care hospitalization) and their caregivers. Using a modified nominal group technique approach, we conducted a series of hybrid in-person/virtual discussions covering three clinical trial topic areas: (*1*) consent and recruitment, (*2*) intervention delivery, and (*3*) trial outcomes. Participants voted on their top preferences in each topic area, and discussion transcripts were analyzed inductively using conventional content analysis.

**Results:**

Thirteen individuals (11 patients, two caregivers) participated in the workshop. For consent and recruitment, participants prioritized technology enabled prescreening and involvement of family members in the consent process. For intervention delivery, participants prioritized measures to facilitate ease of intervention administration and return visits. For trial outcomes, participants identified kidney-related and other clinical outcomes (*e.g*., AKI, CKD, cardiovascular events) as top priorities. Analysis of transcripts provided insight into care team and family involvement in trial-related decisions, implications of allocation to a placebo arm, and impact of participants' experiences of AKI and critical illness.

**Conclusions:**

Findings from our workshop will directly inform development of a clinical trial protocol of cilastatin for nephrotoxic AKI prevention and can assist others in patient-centered approaches to AKI trial design.

## Visual Abstract

**Figure:**
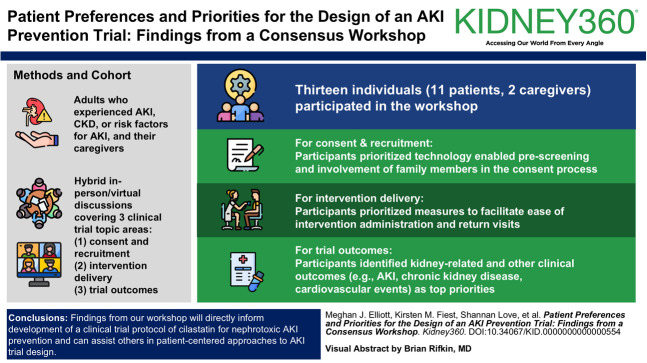

## Introduction

People living with chronic diseases frequently require hospitalization, where they are exposed to a variety of medications,^1–4^ some of which can cause AKI.^5–7^ The consequences of AKI include prolonged hospitalization, CKD, cardiovascular events, and death,^6,8,9^ leading to acute and long-term chronic disease care needs.^10^ No pharmacologic therapies are currently available for prevention of nephrotoxic AKI.^5^

Uptake of nephrotoxins in the proximal tubules of the kidneys is a major contributor to the pathogenesis of AKI.^11–14^ A small molecule called cilastatin can prevent tubular drug uptake and kidney injury through its inhibitory action on two proteins (dipeptidase-1 and megalin).^15–17^ Cilastatin prevents kidney injury in cell culture and animal models of nephrotoxic AKI, and human trials suggest it could be nephroprotective on the basis of clinical studies of imipenem-cilastatin.^18^ In a systematic review, results from ten studies showed lower risks of AKI among patients treated with imipenem-cilastatin compared with inactive or active controls.^19^ A well-designed trial is needed to establish the efficacy of cilastatin alone for this indication.

The perspectives of people with lived experience of a health condition are being increasingly integrated into health research.^20^ Early patient engagement can enhance research feasibility and relevance^21^ resulting in more patient-centered recruitment practices, informational materials, and outcome selection; improved experiences for research participants; and greater adherence to trial interventions.^22–24^ Integrating patient preferences into the design of interventional trials for AKI prevention may make these trials more relevant and responsive to patient needs. We undertook a consensus workshop with people with lived experience of AKI or risk factors of AKI to identify preferences and priorities related to recruitment, intervention delivery, and outcomes for a clinical trial of cilastatin to prevent nephrotoxic AKI.

## Methods

### Study Design and Setting

We held a half-day hybrid in-person/virtual workshop in December 2023 at the University of Calgary. Two thirds of participants attended in person and one third attended virtually using the Zoom platform. We used a modified nominal group technique,^25^ an accepted consensus building approach, to generate and prioritize preferences related to the design of a clinical trial of cilastatin for nephrotoxic AKI prevention among people with lived experience of AKI or risk factors of AKI. During the workshop, three vignettes (*i.e*., clinical scenarios involving fictional patients and caregivers) were used to help guide discussions related to three key aspects of clinical trial design: recruitment and consent, intervention delivery, and outcomes (Supplemental Table 1). This study was approved by the Conjoint Health Research Ethics Board at the University of Calgary (REB23-1564).

### Participants and Recruitment

We recruited adult participants who were comfortable communicating in English and who had either experienced or cared for someone with AKI, CKD, and/or risk factors of nephrotoxic AKI. We purposively sampled participants from nephrology and critical care patient advisory groups in Alberta, Canada, and among participants from related research expressing interest in being contacted about future studies. Research team members distributed email invitations to potential participants and responded to those indicating interest with additional information. A total of 13 participants provided written informed consent before workshop commencement, which is an acceptable sample size for modified nominal group technique studies.^25^

Participants were provided with packages by email before the workshop that included a summary of the topic, the workshop agenda, three vignettes, a consent form, and instructions for use of the virtual platform, if required. In the topic summary, definitions and examples of areas for discussion (*i.e*., recruitment/consent, intervention delivery, and outcomes) were provided. We asked participants to review the vignettes in advance of the workshop and reflect on how a clinical trial in AKI could be designed with the example patients' and caregivers' needs in mind.

### Data Collection

**Figure 1:**
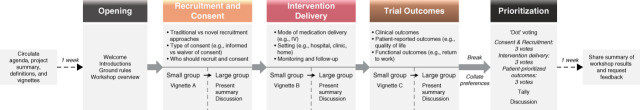
**Overview of phases and flow of the consensus workshop.** IV, intravenous.

An overview of workshop activities is provided in Figure 1. First, one facilitator (M.T. James) welcomed participants and provided a program overview. One virtual and two in-person small groups were established, each with 4–5 participants, an experienced facilitator (M.T. James, M.J. Elliott, and K.M. Fiest), and a note-taker (E. Benterud, S. Love, and B. Rana). Each group participated in three separate discussions covering each topic area of trial design—(*1*) consent and recruitment, (*2*) intervention delivery, and (*3*) outcomes. With reference to a topic guide (Supplemental Table 2) and clinical vignettes (Supplemental Table 1), facilitators ensured participants had opportunity to contribute by directly inviting them to share their thoughts, redirecting the flow of the conversation, and refocusing the discussion around the vignette when required. Following each small group, a facilitator or group participant summarized key points of discussion for the larger group. Before the final prioritization exercise, research team members consolidated and categorized preferences within each topic area.

Using cumulative dot voting,^26,27^ participants were asked to vote on three preferences that they considered most important under each of the topic areas (for a total of nine dots per participant). Participants voted by either placing a physical dot beside their choice (in person) or by selecting their preferred options using the Zoom polling feature (virtual). All discussions were audio recorded and transcribed verbatim. One week after the workshop, participants were invited by email to provide their feedback on the workshop in an evaluation survey (Supplemental Table 3).

### Data Analysis

We summarized demographic data provided by participants descriptively. Preferences were ranked in each topic area by tallying the total number of votes and ranking results as high (≥7 votes), medium (3–6 votes), or low (<3 votes) priority. Priority categories were determined based on the number of workshop participants, available selections within each topic area, and results of other similar consensus-based exercises.^28,29^ The results from the postworkshop evaluation were summarized descriptively (Supplemental Figure 1).

Transcripts from the small-group and large-group discussions were reviewed and inductively analyzed to elaborate on the prioritization results and insights raised during group discussions. Using conventional content analysis,^30^ three research team members (M.J. Elliott, S. Love, and B. Rana) reviewed the transcripts independently and discussed them as a group to generate a list of relevant codes (*i.e*., descriptive labels assigned to segments of text that represent distinct ideas raised by participants). We applied codes systematically across transcripts, reviewed summaries of the data captured by each code, and explored relationships between codes. We then organized codes into higher-level categories, or key concepts, within each of the three topic areas. The key concepts were refined further through discussion among the broader research team and review of handwritten field notes. We ensured methodological rigor through our transparent and reflexive approach to data collection and analysis, systematic application of consensus-based methods with experienced facilitators, researcher and data triangulation, and provision of rich descriptions to support our findings.^31^

### Patient Engagement

Two patient partners (D. Birdsell and M. Loth) with lived experience of AKI and/or CKD were part of the core research team supporting development of the cilastatin trial protocol. Both collaborated on the design, conduct, interpretation, and reporting of this project and participated in the workshop. Another patient partner (H. Dumka) was the colead of the Nephrology Research Group's Patient and Community Partnership at the University of Calgary and helped develop and coordinate the consensus workshop. Patient partners reviewed final outputs and contributed to manuscript preparation. We shared a graphical summary of the prioritization results and thematic findings with all workshop participants 1 month after the workshop and invited them to provide feedback, offer alternative interpretations, and request clarification. We have reported this work in accordance with the Guidance for Reporting Involvement of Patients and Public (Supplemental Table 4).^32^

## Results

Thirteen people participated in the workshop, including four with prior AKI, seven with CKD, six with conditions putting them at risk of AKI (*e.g*., cardiovascular disease, diabetes, nephrotoxic medication exposure), and two with experience of caregiving for a person with AKI or CKD (Table 1). Seven participants (54%) identified as women, seven (54%) were older than 65 years, and 7 (54%) were retired. Most participants (69%) resided in an urban location. Reduced kidney function (*i.e*., eGFR of 30–60 ml/min per 1.73 m^2^) at the time of the workshop was reported by 7 (54%) participants, either among themselves or the corresponding patient for caregiver participants; 2 (15%) patients had received a prior kidney transplant. Five (38%) participants had experience with dialysis treatments, including two (15%) who had received acute dialysis during a critical care hospitalization. In the following sections, we summarize results from the prioritization exercise and key concepts arising from small-group and large-group discussions in relation to identified priorities (Tables 2 and 3).

**Table 1:** Demographic and clinical characteristics of participants (*N*=13)

Characteristic	*No.* (%)
**Condition^a^**	
Person with previous AKI	4 (31)
*Cause of AKI*	
Critical illness (*e.g*., sepsis, critical care stay)	3
Nephrotoxic medication exposure	1
Person with CKD	7 (54)
*Cause of CKD*	
Nephrotoxic medication exposure	2
Prior AKI	1
GN	1
Diabetes	1
Other (*e.g*., reflux, polycystic kidney disease)	2
Person with condition putting them at risk of AKI	6 (46)
*Risk factor*^a^	
Hypertension	3
Hospitalization with critical illness	2
Cardiovascular disease	1
Diabetes	1
Other (*e.g*., nephrotoxic medication exposure)	1
Caregiver of a person with AKI or CKD	2 (15)
**Place of residence**	
Calgary	9 (69)
Edmonton and Northern Alberta	2 (15)
Prefer not to answer	2 (15)
**Age, yr**	
45 or younger	2 (15)
46–55	1 (8)
56–65	3 (23)
66–75	4 (31)
Older than 75	3 (23)
**Education**	
High school	2 (15)
College or trade school	2 (15)
University degree	5 (38)
Professional or graduate degree	3 (23)
Prefer not to answer	1 (8)
**Employment status^a^**	
Retired	7 (54)
Part-time or casual	2 (15)
Full-time	1 (8)
Other (*e.g*., home duties)	1 (8)
Prefer not to answer	2 (15)
**Gender identity**	
Woman	7 (54)
Man	6 (46)
**Languages spoken^a^**	
English	13 (100)
Other (*e.g*., Dene, Italian)	3 (23)
**Self-reported race/ethnicity**	
Black African	1 (8)
Indigenous	1 (8)
White	11 (85)
**Marital status**	
Married	8 (62)
Single	2 (15)
Divorced	1 (8)
Widowed	1 (8)
Prefer not to answer	1 (8)
**Current kidney function^b^**	
eGFR >60 ml/min per 1.73 m^2^	3 (23)
eGFR 30–60 ml/min per 1.73 m^2^	7 (54)
Kidney transplant recipient	2 (15)
Unsure	1 (8)
**Time with kidney disease, yr^b^**	
Less than 5	3 (23)
10–20	4 (31)
More than 20	2 (15)
Unsure or not applicable	4 (31)

a Some participants selected more than one option.

b For patient participants or the corresponding patient with kidney disease for caregiver participants.

**Table 2:** Preferences within each topic area and corresponding prioritization results

Preferences Discussed within Each Topic Area	Vote Count	Priority Rank^a^
**Recruitment and consent process**		
Technology use for recruitment by health care team	11	High
Consent provided by family member	8	High
Multiple methods for consent (*e.g*., one-on-one discussion, visual materials [posters, videos, *etc.*])	7	Medium
Informed consent with knowledgeable and trusted person	6	Medium
Technology use for recruitment by research team	4	Low
Informed consent provided by responsible physician	4	Low
**Intervention delivery**		
Acceptability of a new IV cannula if needed	10	High
Support and reimbursement for return visits to receive intervention	9	High
Intervention duration of more than 1 wk	7	Medium
Intervention administration only during hospital admission	5	Medium
Acceptability of return visits after discharge to receive additional intervention doses if needed	5	Medium
Intervention duration of 1 wk or less	1	Low
**Trial outcomes**		
Short-term and long-term measures of kidney function (*e.g*., serum creatinine, need for dialysis, AKI severity)	10	High
Other patient health complications (*e.g*., cardiovascular events, death)	8	High
Health care utilization (*e.g*., hospital readmission, emergency department visits, length of stay, nephrology follow-up)	5	Medium
Mental health outcomes (*e.g*., anxiety, depression)	5	Medium
Drug-related adverse events	4	Low
Activities of daily living, independence, and functional status (*e.g*., return to work)	4	Low
Physical health, symptoms, and patient-reported outcomes	2	Low
Caregiver outcomes (*e.g*., caregiving burden, mental health)	2	Low

IV, intravenous.

a Priority assignment based on number of votes (*i.e*., dots), defined as high (≥7 dots), medium (3–6 dots), and low (<3 dots) priority.

**Table 3:** Thematic summary with key concepts and supporting quotes

Key Concepts	Supporting Quotes
**Recruitment and consent process**	
Communication of study purpose and processes by trusted team member • Health care team members understand patient's condition holistically • Health care team members as an information relay between patient and research team • Conveying the belief that trial participation is appropriate and low risk • Use of accessible language and terminology	I think the more it [trial recruitment] can be someone who's got a relationship with the wife and the patient. I love the idea of the nurse…a clinical relationship, a trusted relationship—*Participant 1, Group A* The person that's doing it [trial recruitment] has to believe in it and be authentic about it—*Participant 2, Group A* If I had to make a decision for my husband, my biggest concern is when he wakes up is he going to say—“Why did you give that to [me]?” I would want to be assured that that decision I'm making is a good one for him—*Participant 2, Group B* I think as long as they have a good bedside manner, I've had some pretty bad ones in hospital. I think you need the right person to sit down and talk to you. Where they come from, it doesn't really matter, they just have to be good at it—*Participant 1, Group C* We talked about an easy way to explain it to the patient so they understand it and the side effects and the risk associated with the drug. It's that simplification of what the effects are and what the risks are going to be—*Participant, Large-group discussion*
Streamlined recruitment through access to electronic health records • Integration within clinical workflows • Consent to access health records implied as a requirement of inpatient care • Avoiding delays in potentially beneficial treatment for people with critical illness • Respect for privacy concerns by limiting the type/amount of health data accessible to research teams	That's not an invasion of anyone's privacy. There is already invasion at your request, you came in the door of the hospital asking for help—*Participant 2, Group A* There should not be a middleman. It should be direct between the patient and the research team, and it should be the health care system flagging it to the researchers that there is a potential match and then going from there—*Participant 2, Group C* I share the hesitancy with the information being shared from specific groups in our community. If Black people would know that researchers could get a hold of them and talk to them and they might have hesitancy because of the history of research of being abusive toward specific groups, racialized groups and they would be like shocked that research has access to their medical information—*Participant 2, Group C* Everyone was in favor of technology enabled [recruitment] by the research team. They wanted the potential to participate to happen as quickly as possible. They didn't want people to having to comb through their medical record to see if they were on medications, they wanted it to happen quickly—*Participant, Large-group discussion*
Family and/or physician involvement in decisions about participation • Consent by family member when patient condition precludes active care participation • Family member assistance with information processing • Physician advocacy for optimal care extends to trial participation • Credibility of and trust in physician advice	I was in a medically induced coma for 5 wk and all the decisions were made by my wife. I know for a fact the only question that she is going to ask is—what's best for my husband?—*Participant 1, Group B* Patients don't know the medical research behind [intervention]. We trust whatever our doctors are telling us is in our best interest—*Participant 2, Group B* I think the argument would be they [physicians] are already making life and death decisions for you, so if they already add on—well there's also this research study that might help a life-or-death situation—*Participant 5, Group B* It's very important to develop an approach for the recruitment and get the consent from a family member. Some people are not conscious when this is going on, so I think a family member is a key point—*Participant, Large-group discussion*
**Intervention delivery**	
Weighing risks associated with intervention components • Appreciating the risks of participation and nonparticipation • Acceptability of a placebo arm • Commitment to trial participation supersedes concerns about minor risks • Disclosure about possible indirect adverse events (*e.g.*, IV site infection, hospital-acquired infection)	There are even different opinions among the health care team about how long it's safe to leave a line [IV access] in each location. Some people say 48-hours, others would say a week—*Participant 2, Group A* I would make sure that they understand that we've been using this [cilastatin] for a long time in the system and it hasn't had any ill effects on anybody. I think that's very necessary—*Participant 1, Group B* I think the doctor should give you the heads up on the drug, give you the option of taking it or not taking it. I mean, at that point if you are sick and you've got kidney failure, what options do you have?—*Participant 5, Group B* If I knew that I could just get a placebo and that I'm going to actually have some kidney function decrease with this medication that I'll be getting for my cancer, I would be really concerned. I've taken part in medication trials… I found out when it was finished that I actually did [receive study drug], but I was so glad because getting the placebo would have put me in danger of losing my kidney after my kidney transplant—*Participant 3, Group C* They also wanted to know the safety risks. The safety profile of cilastatin would make them more or less likely to agree to a longer-term treatment or a second IV port—*Facilitator, Large-group discussion*
Intervention administration and monitoring tied to routine care activities • Complying with study processes to enable participation • Placement of new IV access only if care team deems necessary • Availability of close monitoring in inpatient setting • Convenience of intervention administration while hospitalized	I would think the conditions that are being treated, you would probably already have an IV. I think most of them would be very small percentage that you would be admitted without an IV in—*Participant 2, Group B* When I was in hospital, I didn't feel sick at all. I'm sitting there on an IV and looking for something to do—*Participant 3, Group B* Wouldn't that be the best time to do it [while in hospital]? You are continually monitored. You are hooked up to an IV—*Participant 5, Group B* I would get it done [receive intervention dose] as many times as required—*Participant 4, Group C*
Logistical considerations to enable follow-up • Reimbursement and support for travel, parking, childcare, lost wages, *etc.* • Minimizing unnecessary facility-based visits • Discontinuation of trial drug before discharge	You have to pay for parking here [in hospital] which is outrageous and you have to maybe pay for a babysitter or a person sitter or you might have to give up hours at work—*Participant 2, Group A* Even if you lived in [town] and you are a patient here. If you are going to come back two and three times a day, I mean—*Participant 4, Group A* I would be nervous going home with it [IV access], to be honest—*Participant 2, Group B* Especially if you live alone, you are not feeling well and you don't have a lot of money for taxis, it's an issue—*Participant 1, Group C* I would do anything not to go into a hospital… Another clinic though, I mean at least it would be controlled, it would be smaller. I would feel more comfortable with that. I could mask up and they would be masked—*Participant 3, Group C* Their preference was if they were to get it [cilastatin] after would be not to come back to the hospital itself. That it would be better to be in a clinic somewhere, somewhere removed from the hospital itself—*Participant, Large-group discussion*
**Trial outcomes**	
Emphasis on kidney-related outcomes • Prioritization of averting adverse renal outcomes, such as AKI or need for dialysis • Availability of routinely collected electronic health data to measure short-term and long-term renal outcomes (*e.g.*, serum creatinine, dialysis dependence) • Capture of both adverse (*e.g.*, AKI) and favorable (*e.g.*, AKI recovery) renal outcomes	It should be emphasized how important kidney function is because they are really not taught to do that in medical school and we don't pay enough attention to it—*Participant 2, Group A* The clinical stuff is number one, I would say, to make sure his [patient from vignette] kidney function hasn't decreased—*Participant 3, Group B* They couldn't believe my recovery, and you probably remember how my kidneys went from this [low] to this [high] again, and you said, “Holy Dinah”—*Participant 5, Group B* The one thing that comes into my mind would be the long-term, probably clinical [outcomes]. That you are going to be left with a nonfunctioning or low functioning kidney leading to dialysis or loss of kidney function—*Participant 1, Group C* If you are in some sort of acute scenario keeping you alive, keeping your organs working for a long period of time and not having negative side effects are kind of the most important—*Participant 4, Group C*
Return to an acceptable quality of life and functional status • Re-establishing previous level of physical and mental wel-lbeing • Quality of life is affected by renal and other clinical outcomes • Not overlooking caregiver outcomes, such as caregiving burden and mental health • Objectively measuring of quality of life with validated tools (*e.g.*, patient-reported outcome measures)	I just think, for me [as caregiver], it's overwhelming, the world of ICU… It's a real challenge for family members—*Participant 1, Group A* I can tell you the kidney damage to quality of life is a lot more profound than patients will normally tell you. I mean people look at me and say, well you are ok because I play tennis four times a week and I'm doing almost everything I did before, but it's not the same. I'm not my normal self and I can't live a normal life.—*Participant 2, Group A* I would want to see my husband heal, get better… We want to see him get out of that bed and walk—*Participant 3, Group A* If they were working before they got sick, when they get better they could go back to work. You just want to be able to do the things you did before you got sick—*Participant 2, Group B* Taking care of me is taking care of my physical health and my mental health also. Both work hand in hand with each other. If somebody goes into hospital and they come out and have a mental [health] issue after because of what was done to them in the hospital—that's not right. We've got to protect that with those patients going in and out of the hospital—*Participant 1, Group B* I would think, you know, hoping that he would be able to work and be part of the family would be a big, big thing… I'm thinking long term consequences that would be the most important and that would be one of them—*Participant 1, Group C*
Expectations for monitoring outcomes and ensuring safety • Trends over time (*e.g.*, kidney function, functional status) as important as dichotomous outcomes • Frequency, timing, and specific testing at the discretion of care team • Delegating responsibility for follow-up (*e.g.*, nephrologist versus primary care) • Monitoring for long-term safety of intervention	Whatever they [care team] need to do for a test to determine whether the medication is working or not—*Participant 1, Group A* It would be nice to know who should be in charge of that [monitoring outcomes], the family doctor or a nephrologist?—*Participant 2, Group A* We need to trend those stats… Where they are at, where they are going. Is it better? Is it worse?—*Participant 4, Group A* I think when you are dealing with the spouse, safety first has got to be the key with that—*Participant 1, Group B* When something happens to somebody, I think it's very necessary to measure and say, this is what our expectations are going forward—*Participant 1, Group B*

ICU, intensive care unit.

### Preferences for Trial Recruitment and Consenting Processes

Within the recruitment and consent topic area, participants highly prioritized health care team members' access to electronic health records for identifying and recruiting eligible patients to the trial (11 votes). This included acceptance of a waiver of consent for access to health data for eligibility screening (*i.e*., technology enabled prescreening). Participants also prioritized informed consent by family members of patients who are critically ill and/or unable to provide consent themselves (eight votes).

During group discussions, participants emphasized the importance of rapport and trusted relationships with personnel approaching patients about trial participation and clear communication of what their participation would entail. They preferred being initially approached by a health care provider rather than by the research team directly because of the clinical team's familiarity with the patient, knowledge of the clinical context, and care continuity. Participants favored the use of electronic health records for eligibility screening as a way of streamlining recruitment and avoiding delays in initiating a potentially life-saving therapy. While participants acknowledged privacy concerns, they suggested the benefits of prompt participant identification and recruitment through access to limited electronic health data outweighed these risks. Participants indicated that consent for trial participation should be provided by patients themselves but that informed consent from substitute decision makers or trial enrollment by trusted physicians, who are responsible for life and death decisions for you, would be acceptable in circumstances of critical illness precluding active care participation (*e.g*., sedated, unconscious, ventilated).

### Preferences for Intervention Delivery

In the prioritization exercise, most participants indicated that placement of a new intravenous (IV) access would be acceptable if needed to deliver the trial intervention (ten votes). Participants also identified the provision of support and reimbursement for return trial visits, whether for further intervention doses or for monitoring, as a high priority (nine votes).

Participants from all three small groups raised concerns about the acceptability of the trial's placebo arm without prompting. Given the high stakes of AKI and potential benefits of the intervention, participants indicated it would be important to communicate to patients up-front that they have a 50% chance of receiving cilastatin and why a trial designed in this way is needed to establish its safety and efficacy. Participants prioritized their participation in a trial with the potential to avert AKI over details regarding how cilastatin would be administered and concerns about its minor risks. However, they did indicate that use of an existing IV cannula would be preferable to placing a new one, particularly for patients with difficult peripheral venous access or needle phobia, and that trial medication dosing should be coordinated with other routine care activities. Small-group discussions also covered the convenience of intervention delivery as an inpatient, where patients are continually monitored and hooked up to an IV, and preferences for receiving the intervention drug only while hospitalized or, if required after discharge, through home visits because of concerns about the safety of long-term IV cannulation and logistical challenges of returning to hospital. They also anticipated barriers to surveillance after hospital discharge, such as travel, parking costs, and lost wages, for which they expected the study team would provide support.

### Preferences for Trial Outcomes

Top priorities for trial outcomes included short-term and long-term measures of kidney function (*e.g*., AKI, need for dialysis; ten votes) and other clinical events (*e.g*., cardiovascular events, death; eight votes).

Discussions about trial outcomes centered on averting adverse renal outcomes, specifically preventing AKI, AKI progression, and need for dialysis, and leveraging routinely collected clinical and laboratory data for outcome ascertainment. Kidney and other clinical end points were largely discussed in relation to the complexity of hospitalized patients with AKI and the anticipated negative impact on quality of life and mental and physical health. Although quality-of-life outcomes were not prioritized during the voting exercise, participants discussed attaining one's previous level of functional and mental well-being as an important long-term outcome. They also identified a need to measure quality of life objectively using validated tools (*e.g*., patient-reported outcome measures) and in a way that considers the impact on both patients and caregivers. While they did not express a preference for specific instruments or tests to ascertain outcomes, participants valued trends over time in kidney function, quality of life, and functional status. Participants also raised concerns about the long-term safety of cilastatin and suggested that defining expectations going forward for monitoring over the course of the trial, including timing, responsible care team members, and long-term safety, would reassure those considering enrolling in the trial.

### Evaluation Survey

All participants (*N*=13) completed a postworkshop evaluation survey (Supplemental Figure 1 and Supplemental Table 3) and indicated the workshop goals were communicated clearly and the materials were presented in an organized, well-paced way. Eleven participants stated that their opinions were captured in the large-group report-back summaries, and all but one participant felt the final voting results reflected the opinions and preferences discussed during the workshop. Three participants did not feel the vignettes added value to the workshop, with one suggesting they may have detracted from discussion about the experiences of the individual patient and caregiver participants.

## Discussion

In this consensus workshop for the design of an AKI prevention trial, participants identified priorities that included the use of technology (*i.e*., access to electronic health records) and involvement of trusted individuals in trial recruitment, logistics of intervention administration (*i.e*., IV access, during hospitalization), support for study follow-up, and emphasis on kidney-related and quality-of-life outcomes. While this workshop centered on the proposed trial intervention, cilastatin, our findings can also help other trials for AKI develop patient-centered approaches to recruitment and consent processes, intervention delivery, and outcome selection.

Low accrual rates and delays in identifying eligible patients put the viability of clinical trials at risk, and both are common when research staff must manually find participants for trials. Technology enabled prescreening is an increasingly accessible way to ensure systematic and timely identification of potential participants for modern-day trials as the availability of digital clinical information systems expands.^33^ Although privacy legislation governs how health data can be accessed, patient perspectives on use of such digital approaches may affect the willingness of patients to participate in trials. Technology enabled prescreening is particularly relevant to trials seeking to enroll people with or at risk of AKI because this population is widely distributed across hospital units and clinical services, making traditional manual approaches inefficient.

Participants' prioritization and acceptance of technology enabled prescreening is consistent with other research suggesting that patients are willing to share their health information digitally provided they are clear on the rationale, how privacy will be assured, and the value of the research.^34,35^ A recent national survey in Canada found that most respondents preferred data sources to be accessible by health care providers and delegates as the default option.^36^ Because acceptability of recruitment and consent models has been identified as an important driver of critical care trial success,^37^ involving patients in designing consent processes and exploring alternative consent approaches may further address barriers to trial participation in critical care contexts.^38,39^ For example, consent from a substitute decision maker may be appropriate when patients are unable to provide informed consent themselves because of critical illness.^39^ Deferred consent is another approach to address ethical precepts of informed consent under emergency circumstances or where a substitute decision maker is unavailable. Our workshop participants perceived these approaches as acceptable for a trial in AKI with a narrow recruitment window and identified the physician as a knowledgeable, trustworthy person in the circle of care responsible for communicating with substitute decision makers or enacting deferred consent processes as appropriate. Taken together, our findings and those of others imply patient support for the use of technology enabled prescreening for clinical trials in AKI when the value of access to the information for trial success is clear, privacy and security concerns are addressed, and person-centered consent processes are followed.

Participants in our workshop were willing to comply with processes necessary for administering the intervention if recommended by research and care teams. However, they made suggestions for integrating trial intervention delivery with other routine care activities while in hospital to minimize burdens of trial participation, such as need for additional testing and/or return visits, financial challenges, and concerns about safety.^40,41^ Embedding trial processes within clinical workflows, such as timing cilastatin administration with other medications or coordinating laboratory tests with routine inpatient bloodwork, could further reduce the burden for health care team members.^42^ Concerns raised by participants about allocation to a placebo arm when the cilastatin intervention might help prevent AKI-associated outcomes align with patients' perspectives on trial design from a qualitative study by Gaasterland *et al.*^43^ Because patients view a novel intervention as a source of hope, the possibility of not receiving a potentially beneficial intervention may compromise this hope, the perceived benefits of trial participation, and ultimately patients' willingness to enroll in randomized trials.^43^ While participants in our study sought assurances of benefit from participating, they acknowledged that the process of randomization meant a 50/50 chance of receiving a new treatment with uncertain efficacy and that trial participation entailed creating new knowledge to inform future care more so than conferring benefit to themselves directly. In acknowledging these perspectives early in the design phase, recruitment materials and communication strategies can be codeveloped with patients to clearly articulate trial processes intended to establish intervention efficacy and safety.

Several narrative reviews have been published on the selection of outcomes for AKI trials, although these papers have focused on methodological aspects of outcome measures and requirements for regulatory drug approval.^44–49^ Although reports have called for greater patient participation in the design of trials for AKI,^45^ few studies have explored patient priorities for AKI trial outcomes. The Standardised Outcomes in Nephrology initiative has established core outcome measures across the spectrum of kidney disease.^50^ Although the Standardised Outcomes in Nephrology initiative has not addressed outcomes for AKI trials, findings from a focus group study among patients and care providers of people with CKD may be relevant to trials for AKI, including the high priority assigned to outcomes of kidney function, mortality, fatigue, life participation, and mental health.^51^ Another report from a workshop focused on improving care for patients after hospitalization with AKI included three patients and reported that symptom burden coupled with uncertainty about recovery of kidney function exerted a psychological toll on patients.^52^ Similar to these reports, we found that patients prioritized measures of kidney function, both short and long terms, as the top-ranked trial outcome, followed by clinical outcomes including survival and cardiovascular events for AKI trials. Notably, our findings from patients align with recent recommendations from AKI trialists to consider the occurrence of AKI as a key end point for phase 2B prevention or attenuation trials and major adverse kidney events (including death, dialysis, or a sustained reduction in kidney function) for phase 3 AKI prevention, attenuation, or treatment trials.^44–46^ While our participants gave lower relative weightings to mental and physical function outcomes, prevention of AKI and avoidance of dialysis were described by patients as important contributors to quality of life and thus remained relevant trial outcomes.

Our study is strengthened by the involvement of people with lived experience in workshop organization and the capture of diverse insights related to AKI, including participants with critical illness and AKI experience, as well as those with risk factors for or sequelae of AKI (*e.g*., CKD, dialysis dependence). However, we acknowledge some limitations. First, the time allotted for small-group discussions may have been insufficient for participants to reflect and elaborate fully on important experiences, and some participants may have felt uncomfortable sharing their perspectives in this forum. We used skilled facilitators to encourage respectful interactions, explain the rationale for clinical trials and key concepts (*e.g*., equipoise, uncertainty of risks/benefits), and redirect the flow of conversation to ensure all participants had the opportunity to contribute ideas about trial participation. Second, the priorities brought forward to the voting exercise were compiled in real time following small-group discussions, which means that preferences expressed by one participant or not discussed at length may not have been captured among voting options. However, results from the workshop evaluation survey suggest that participants felt the outcome reflected the content of group discussions. Third, our study participants, who were largely White, cisgender, and highly educated, may have been more accepting of the risks of research and may have held views that differ from those of underrepresented groups at risk of AKI. Although discussions did cover aspects of socioeconomic disadvantage and health inequity (such as costs incurred for outpatient visits and hesitancy to participate in research among racialized groups), this area warrants future dedicated study. Finally, the hybrid format may have limited participation by virtual attendees, although this may be outweighed by the inclusivity and diversity of participation enabled by the hybrid approach.

Patients and caregivers prioritized technology enabled prescreening and integration of trial processes and intervention delivery with routine care activities to facilitate participation in a clinical trial of cilastatin for preventing nephrotoxic AKI. Participants' prioritization of kidney-related and other clinical end points related in large part to their desire to avoid sequelae of AKI, such as dialysis dependence, and restore physical and mental well-being after hospitalization. These perspectives will inform development of an AKI prevention trial protocol and can also help others develop patient-centered approaches for recruitment and consent, intervention delivery, and outcome selection for AKI trials.

## Supplementary Material

SUPPLEMENTARY MATERIAL

## Data Availability

All data are included in the manuscript and/or supporting information.

## Supplemental Material

Supplemental Table 1. Vignettes.

Supplemental Table 2. Workshop topic guide.

Supplemental Table 3. Postworkshop evaluation.

Supplemental Figure 1. Summary of findings from postworkshop evaluation.

Supplemental Table 4. Guidance for reporting involvement of patients and public (GRIPP2) short form.
